# The role of N6-methyladenosine (m^6^A) in kidney diseases

**DOI:** 10.3389/fmed.2023.1247690

**Published:** 2023-09-28

**Authors:** Luling You, Zhongyu Han, Haoran Chen, Liuyan Chen, Yumeng Lin, Binjian Wang, Yiyue Fan, Meiqi Zhang, Ji Luo, Fang Peng, Yue Ma, Yanmei Wang, Lan Yuan, Zhongyu Han

**Affiliations:** ^1^School of Medical and Life Sciences, Chengdu University of Traditional Chinese Medicine, Chengdu, China; ^2^Science and Education Department, Chengdu Xinhua Hospital, Chengdu, China; ^3^Eye School of Chengdu University of Traditional Chinese Medicine, Chengdu, China; ^4^School of Medical Information Engineering, Chengdu University of Traditional Chinese Medicine, Chengdu, China; ^5^School of Clinical Medicine, Southeast University, Nanjing, China; ^6^Institute of Traditional Chinese Medicine, Sichuan College of Traditional Chinese Medicine (Sichuan Second Hospital of TCM), Chengdu, China

**Keywords:** RNA modification, m^6^A, kidney diseases, acute kidney injury, chronic kidney diseases, renal cancer

## Abstract

Chemical modifications are a specific and efficient way to regulate the function of biological macromolecules. Among them, RNA molecules exhibit a variety of modifications that play important regulatory roles in various biological processes. More than 170 modifications have been identified in RNA molecules, among which the most common internal modifications include N6-methyladenine (m^6^A), n1-methyladenosine (m^1^A), 5-methylcytosine (m^5^C), and 7-methylguanine nucleotide (m^7^G). The most widely affected RNA modification is m^6^A, whose writers, readers, and erasers all have regulatory effects on RNA localization, splicing, translation, and degradation. These functions, in turn, affect RNA functionality and disease development. RNA modifications, especially m^6^A, play a unique role in renal cell carcinoma disease. In this manuscript, we will focus on the biological roles of m6A in renal diseases such as acute kidney injury, chronic kidney disease, lupus nephritis, diabetic kidney disease, and renal cancer.

## Introduction

The kidney functions as an essential excretory device adept in the intricate regulation and maintenance of the physicochemical milieu of the human body. By finely tuning the balance of water, electrolytes, and other vital substances, this organ elegantly stabilizes the internal environment of the body and could rightfully be considered a linchpin of homeostasis (1). Severe or persistent kidney damage often leads to tubular degeneration, inflammation, renal fibrosis, and ultimately chronic kidney disease (CKD) or end-stage renal disease (2). It is well known that both acute kidney injury (AKI) and CKD have become important clinical problems and global public health issues, affecting more than 750 million people worldwide (3). It is estimated that 17 million hospitalized patients experience AKI each year, with a significant increase in mortality, length of stay and the development of other complications (4). The population prevalence of chronic kidney disease exceeds 10% and in high-risk subpopulations exceeds 50% (5). Kidney diseases pose a serious burden on global health as well as on healthcare systems.

A growing number of studies suggest that RNA modifications play an integral role in the development of renal diseases. RNA modifications are chemical modifications that occur at different atoms of bases and are of numerous types; more than 170 chemical modifications have been identified on RNA to date, and more than half are methylation modifications. The distribution sites involve a variety of RNAs, including messenger RNA (mRNA), non-coding RNA (ncRNA), and different types of bases (A, C, G, U) (6–8). Highly dynamic RNA modifications play an important role in regulating RNA fate and are fundamental mechanisms for regulating the cellular transcriptome and proteome (9).

**Figure 1:**
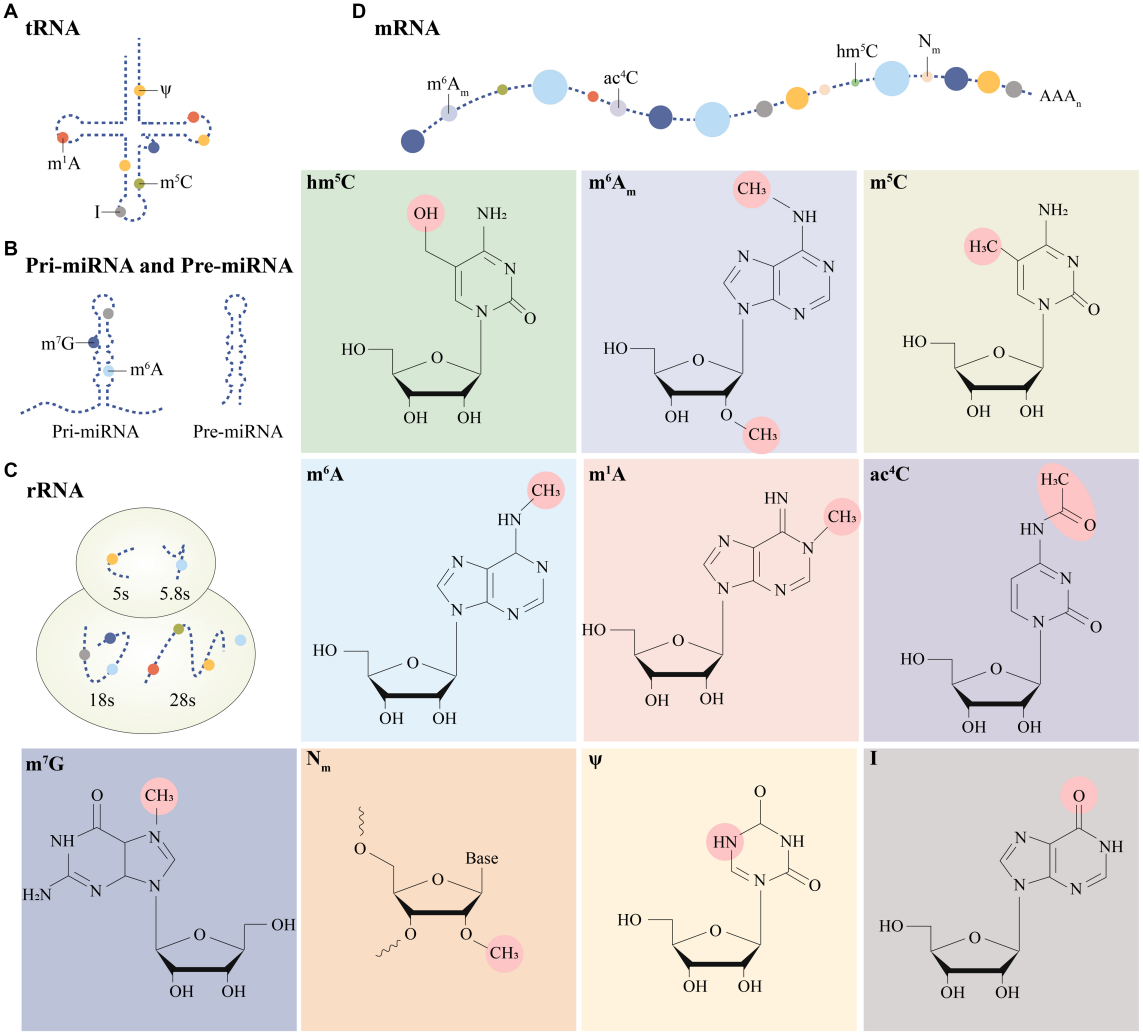
Distribution of multiple post-transcriptional modifications on different RNA isoforms **(A–D)**. Specific modification moieties are prominently labeled in post-transcriptional modifications.

**Figure 2:**
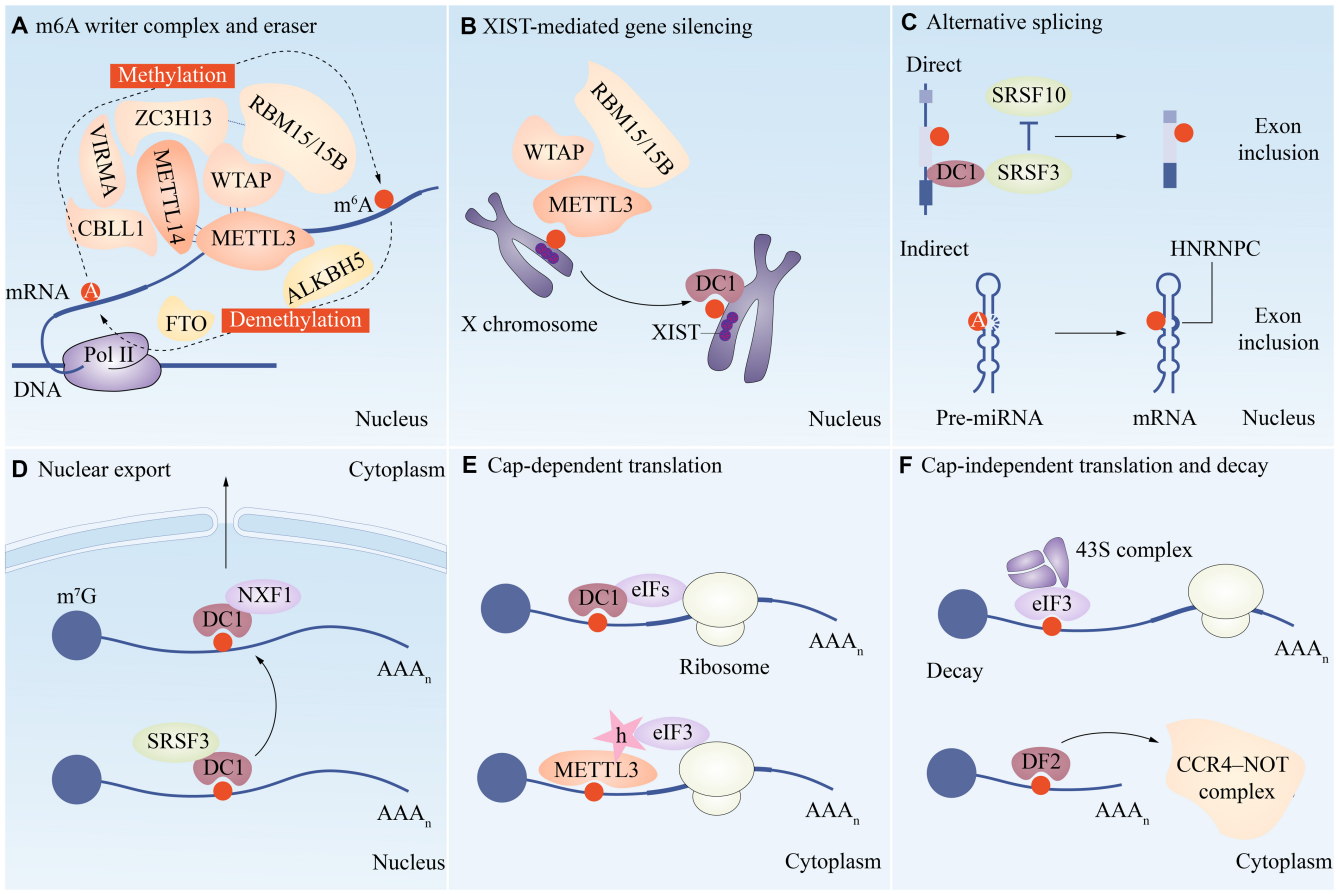
A functional overview of m^6^A modifications during the RNA life cycle. **(A)** METTL3, METTL14, WTAP, VIRMA, RBM15/15B, CBLL1 and ZC3H13 are combined to form the writer complex to catalyze the methylation of m^6^A. The demethylase FTO and ALKBH5 are the m^6^A eraser. Several m^6^A reader proteins are involved in regulating multiple aspects of mRNA metabolism, such as XIST-mediated gene silencing **(B)**, alternative splicing **(C)**, nuclear export **(D)**, translation **(E/F)**, and decay **(F)**.

Many important modifications have been identified on mRNA and ncRNA, such as N6-methyladenosine (m^6^A) modification, N1-methyladenosine (m^1^A) modification, 5-methylcytosine (m^5^C) modification, 7-methylguanosine (m^7^G) modification, etc. (Figure 1). Among them, the study about m^6^A modification is more in-depth, and a review of the literature shows that there are three main types of molecules involved in the modification: methyltransferases (i.e., writing proteins), demethylases (i.e., erasure proteins) and methyl-binding proteins (i.e., reading proteins), which perform catalysis, erasure and recognition, respectively. They act as a complex whole to dynamically regulate RNA localization, splicing, translation and degradation, which in turn affects RNA function and disease (10) (Figure 2).

In the next sections, we will briefly describe the regulatory mechanisms and functions of major RNA modifications in eukaryotic cells, such as m^6^A, m^5^C, m^7^G, and m^1^A. We also highlight the biological and clinical roles of m^6^A modifications in renal diseases, such as AKI, CKD, diabetic kidney disease (DKD), lupus nephritis (LN), and renal cell carcinoma (RCC).

## m^6^A

Methylation at the 5′ Cap is widely recognized as playing important roles in maintaining mRNA stability, precursor splicing, polyadenylation, transport, and translation initiation, and is a common modification found in the vast majority of eukaryotes. In addition, along with poly A binding protein, the changes at 3′ poly A have a role in outgoing nuclear transport, translation initiation, and mRNA structural stability maintenance (11). However only the head and tail of mRNA undergo these modifications, and the majority of these internal changes are m^6^A methylation changes. The term “m^6^A” refers to the dynamic and reversible methylation modification (N6-adenylation) that occurs on the 6th N of RNA adenylate and affects a variety of mRNA metabolic steps, including splicing, translation, nuclear export, and stability (Figure 2). m^6^A has the ability to influence gene expression and thus biological activities such as cell self-renewal, differentiation, invasion, and apoptosis (12).

The biological behavior of m^6^A is powerfully influenced by the concerted action of the write protein, the erase protein, and the read protein. These pivotal proteins collaboratively and intricately sculpt the epitranscriptome, playing a non-trivial role in shaping the phenotypic blueprint of cells, tissues, and organisms. For the m^6^A methyltransferase, which catalyzes the change from A to m^6^A on mRNA, Methyltransferase-like protein 3 (METTL3) and Methyltransferase-like protein 14 (METTL14) form a writing complex (13, 14) (Figure 2). m^6^A binding proteins that identify m^6^A methylation and start functional signaling include YTH m^6^A RNA binding protein 1–3 (YTHDF1-3), YTH domain containing 1–2 (YTHDC1-2), and insulin-like growth factor 2 mRNA-binding protein 1/2/3(IGF2BP1/2/3). Fat mass and obesity-associated protein (FTO) and alpha-ketoglutarate-dependent dioxygenase ALKB homolog 5 (ALKBH5), which are two protein demethylases, can remove m^6^A alterations from RNA, reversing and regulating the methylation-dependent process (15).

### Writers

METTL3 was shown to be a crucial component of the m^6^A mRNA methyltransferase complex in the 1990s. Later, it was discovered that METTL14 is one of the complex’s fundamental components (16, 17). In the complex machinery of methyltransferase METTL3, where precision is paramount, the catalytically active center would be incomplete without the indispensable structural scaffold effortlessly provided by METTL14. The tight-knit orchestration between these interdependent proteins is vital to the flawless execution of methyltransferase activity, thereby intricately regulating the epitranscriptomic landscape. Accessory units including Wilms tumor 1-associated protein (WTAP), vir like m6A methyltransferase associated (VIRMA), RNA binding motif protein 15A/15B (RBM15A/RBM15B), zinc finger CCCH-type containing 13(ZC3H13), and HAKAI (a potential E3 ubiquitin ligase) are also present in larger methyltransferase complete complexes (18–21) (Figure 2). Further research revealed that methyltransferase-like protein 16 (METTL16) also functions by itself in mammals as a m^6^A reader protein, although it only methylates a small subset of RNAs with particular RNA structures, such as U6 snRNA (22).

In addition to being a key component of METTL3, which identifies the sequence motif GGACU and employs s-adenosylmethionine (SAM) as a methyl donor to add methyl to its adenosine residues, the writer protein of m^6^ A is primarily responsible for the m^6^A methylation modification of bases on mRNA (23). The METTL14 subunit of the m^6^A methyltransferase complex serves as the complex’s primary supporting component and speeds up m^6^A RNA methylation catalysis (24). WTAP is a regulatory member of the METTL3/METTL14 methyltransferase complex that modulates METTL3/METTL14 complex aggregation to transcriptional and pre-mRNA-processed areas of nuclear scatter (14, 25, 26). The WTAP/METTL3 catalytic complex can be directed to an existing m^6^A site, creating a new m^6^A marker nearby and serving as a recruiter (27).

### Readers

In the intricate choreography of biological processes, the methylation of the mRNAs that undergo m^6^A changes is a crucial and finely orchestrated step, where reading proteins bearing the methyl mark are indispensable. These epitranscriptomic readers perform specific and tailored functions, enabling the mRNAs to execute their biological tasks with utmost precision and efficacy, thereby perpetuating the complex web of life. Eukaryotic initiation factors (eIFs), IGF2BPs, and proteins with the YTH structural region make up the majority of reading proteins. These reading proteins primarily operate by specifically binding to the m^6^A methylation area, reducing homologous binding to RNA-binding proteins, and changing RNA secondary structure, which in turn affects protein-RNA interactions.

The delicately poised phenomenon of mRNA translation is facilitated by an intricate interplay between effectors (molecules that directly cause a specific response or effect in cells) and regulators (molecules control and modulate the activity of effectors or other cellular processes). Among these, YTHDF1, a crucial reader protein, stands out as a master orchestrator, selectively binding to eIF3 and facilitating the translation efficiency of m^6^A-modified RNA targets (28). Transcript breakdown occurs when YTHDF2 co-localizes with proteins from the dead enolase complex and delocalization complex at the p-body of the mRNA attenuation site, where it carries its target mRNA. YTHDF3 aids RNA translation and RNA degradation by interacting with YTHDF1 and YTHDF2, respectively, and building partnerships with YTHDF2 (29–32). IGF2BP is a crucial regulator of targeting RNA translation, stability, splicing, and intracellular localization by direct binding of its KH structural domain to m^6^A RNA and targeting mRNA transcripts by identifying the consistent GG(m^6^A)C sequence. Under usual conditions, enhances target mRNA stability by interacting with mRNA regulators including ELAVL1 and matri3 (33).

### Erasers

Within the intricate landscape of epitranscriptomic modifications lies an important player, the eraser protein, which functions as a demethylase, skillfully plucking off the methyl group borne by m^6^A with the aid of ferrous iron, a coenzyme, and α-ketoglutarate, a co-substrate. This finely optimized interplay between enzyme and substrate underscores the delimited precision with which epigenetic regulation is governed, illuminating the remarkable intricacies that lie beneath the surface of molecular biology (34). The primary m^6^A demethylases are FTO and ALKBH5, among others.

The FTO protein, also known as fat mass and obesity-associated protein, is a member of the ALKB protein family and has been related to obesity (35). The University of Chicago group led by Professor Chuan He made the initial discovery that the FTO protein is a substantial demethylating enzyme in 2011 (36). ALKBH5, another essential demethylating enzyme, alters mRNA by demethylating it in the nucleus (37). The level of m^6^A modification on mRNA significantly increased when the cell line’s ALKBH5 was knocked down.

### Distribution and dynamic regulation of m^6^A modifications

At the core of the complex and fascinating landscape of epitranscriptomics lies m^6^A, the most abundant and widespread chemical modification that adorns the mRNA skyline. As detection technologies have evolved, an impressive array of RNA species, including but not limited to transfer RNA (tRNA), ribosomal RNA (rRNA), small nuclear RNA (snRNA), and diverse non-coding RNAs (ncRNAs), have been reported to harbor this ubiquitous modification, underscoring the vital and far-reaching implications of this epigenetic alteration and its role in the myriad molecular interactions that govern the intricate machinery of life (38)(Figure 1). According to research, m^6^A modification is the most prevalent internal alteration linked to eukaryotic mRNAs and is involved in practically all stages of mRNA metabolism, including splicing, export and translation, and mRNA breakdown (Figure 2).

m^6^A is asymmetrically distributed on mRNA, and it is usually clustered near the stop codon, the 3′ untranslated region (3’UTR), and the long internal exon, with some occurring in the 5′ untranslated region (5 ‘UTR) and at the transcription start site. Only a small number of RNAs have both m^6^A modifications in the 5’ UTR region, CDS region, and 3′ UTR region. m^6^A has a conserved modification motif RRACH (R for A or G, H for A, U, or C) and methylation occurs at the sixth nitrogen atom of adenine, and this modification is dynamic and reversible (39).

m^6^A modifications play a crucial role in RNA splicing. It has been shown that m6A modification can regulate splicing factors binding to RNA, affecting splicing position and splicing efficiency. Splicing is the step of removing introns and joining exons after RNA transcription. Pre-mRNAs must undergo 5′ end and 3′ end modifications (5′-capping, 3′-polyadenylation) as well as splicing to form mRNAs in eukaryotic cells. Besides, pre-mRNA introns have many m6A sites, which are more numerous than in mature mRNAs (40).

As mentioned above, METTL3, which is the catalytic subunit in Writer, is localized to nuclear patches enriched in mRNA splicing factors, particularly concentrated on mRNA, which undergoes selective splicing, suggesting a potential regulatory role for m^6^A in mRNA splicing. It has been shown that m^6^A modification of transcripts is altered when METTL3 is absent, leading to abnormal splicing of important spermatogenic regulatory genes such as Sohlh1 and Dazl (41). WTAP deletion can also lead to altered mRNA isoforms that significantly reduce the ability of METTL3 to bind to RNA, thereby affecting mRNA splicing (18).

The m^6^A Reader affects mRNA splicing as well. As a nuclear m^6^A reader, YTHDC1, one of the Reader’s constituent proteins, binds to m^6^A directly. Moreover, it interacts with splicing regulators including SRC associated in mitosis of 68 Kd (SAM68), splicing component 35(SC35), and Serine/arginine-Rich Splicing Factor 1/3(SRSF1/3), to which YTHDC1 may attach following mRNA transcription and methylation. In order to enable splicing or other nuclear activities, m^6^A-modified mRNAs are recruited by the low-complexity structural domain of YTHDC1 in certain nuclear structures (15). These results suggest a possible connection between YTHDC1 and mRNA splicing.

Ultimately, a variety of eraser parts have comparable functions throughout the splicing process. FTO can demethylate m^6^A_m_ and cells knocked out of FTO display a splicing defect, probably due to the fact that the physiological substrate of FTO is snRNA, which mediates splicing, and m^6^A_m_ in snRNA may be altered thereby causing the splicing process to not proceed properly (15, 42). AlKBH5 is an endogenous m^6^A demethylase since its overexpression and knockdown cause cells to produce more and less m^6^A, respectively. When ALKBH5 is absent, the staining of alternative splicing factor/splicing factor 2 (ASF/SF2) is significantly diminished in HeLa cells and the nuclear scatter localization of numerous splicing factors is affected indicating that ALKBH5 also plays a role in the pre-mRNA splicing process. Hyperphosphorylated ASF/SF2 can be involved in pre-mRNA splicing (18).

By binding to ribosomal subunits, m^6^A modification promotes RNA translocation and export, and participates in intracellular RNA localization and regulation. After splicing is completed, mature mRNA is transported through the nuclear pore to the cytoplasm. In contrast, METTL3, ALKBH5, and YTHDC1-mediated m^6^A can affect nuclear processing and export of mRNA, thereby selectively regulating gene expression. Deletion of METTL3 inhibits mRNA export, whereas deletion of ALKBH5 enhances mRNA export to the cytoplasm (43, 44).

During translation initiation, m^6^A modifications can exert an influence on the structure and function of RNA molecules involved in translation, which in turn regulate gene expression levels. The academicians have postulated three primary translation upregulation mechanisms connected to m^6^A that occur during mRNA translation. Originally, YTHDF1 recruits translation initiation factor eIF3 and binds to m^6^A-modified mRNA to begin and increase translation of m^6^A-modified mRNA (45). The second pathway involves the direct interaction of eIF3 and the 5′ UTR m^6^A. The presence of m^6^A on the 5′ UTR enhances cap-independent translation, and eIF3-m^6^A association also facilitates ribosome loading (46). The penultimate one is METTL3 directly activating translation (18, 47). The precise method is still being studied, however after mRNA enters the cytoplasm, METTL3 binds eIF3 and interacts with mRNA cap-associated proteins (48).

Lastly, m^6^A modifications have the potential to regulate the stability and lifespan of RNA molecules by impacting the post-transcriptional degradation mechanism. Among them, reading proteins, primarily YTHDF2 and YTHDF1, play a major role in the degradation of mRNA. Similar to YTHDF1, YTHDF2 is a protein with two structural domains that seems to hasten the breakdown of n6-adenosylmethylated mRNAs by directly attracting the carbon catabolite-repression 4-Not (CCR4-Not) complex to the substance (49). The target mRNA becomes more stable when YTHDF2 is decreased (50). In human and mouse cells, downregulation of the m^6^A writing protein (METTL3 or WTAP) results in an increase in the half-life of mRNA (51). Each component of the m^6^A erasure, writing, and reading proteins has a particular role at various points during the mRNA maturation and cleavage process.

## m^6^A in kidney diseases

m^6^A, the most prevalent RNA modification, has been shown to exert its function in multiple ways, including splicing, export, decay, and translation initiation efficiency, to regulate mRNA fate. RNA modifications have been shown to be heavily involved in kidney development and the progression of disease. Additionally, it has been noted that m^6^A affects biological processes by upsetting stable base pairing, which controls a number of RNA functions. Most notably, it has been discovered that m^6^A is linked to a growing number of kidney disorders, including RCC, acute kidney injury, and chronic kidney disease. The RNA demethylase FTO is currently referred to be abundant in the kidney and governs the fibrotic process in obstructive nephropathy through the TGF-β signaling pathway, according to related studies (52). The degree of renal interstitial fibrosis was found to be strongly correlated with the m^6^A methylation alterations by Li et al. (53). We will next focus on the key role played by m^6^A in AKI, CKD, DN, LN, and RCC, as well as the related pathogenesis.

### Renal immune microenvironment

**Figure 3:**
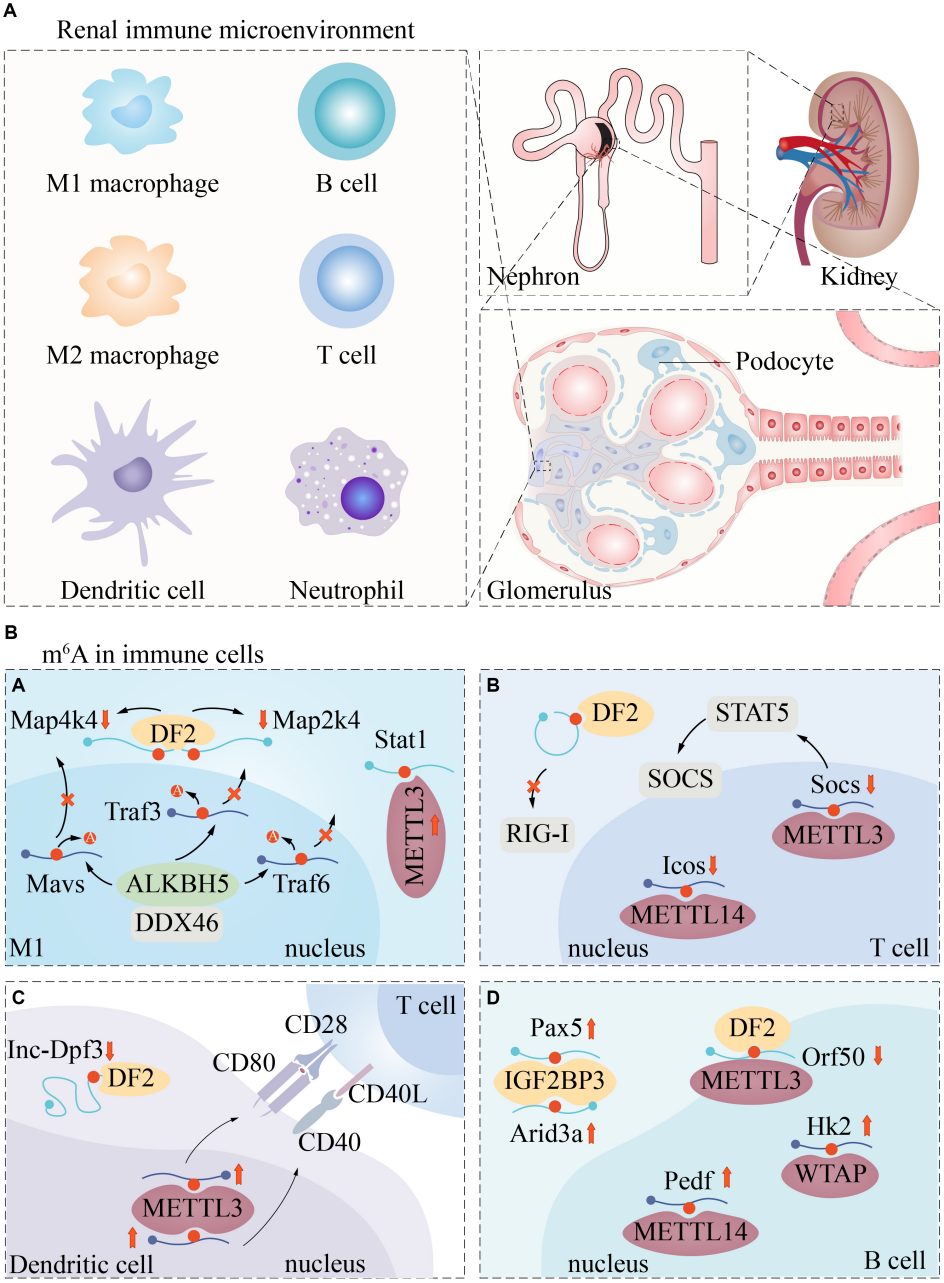
Renal immune microenvironment and m^6^A regulatory pathways in immune cells. The renal immune microenvironment includes but is not limited to these immune cells **(A)**. Currently, m^6^A modification has been poorly reported in immune cells. We described the regulatory expression of m^6^A -related proteins in macrophages **(Ba)**, T cells **(Bb)**, Dendritic cells **(Bc)**, and B cells **(Bd)**.

The kidney is a complex organ of great functional importance that plays a critical role in removing toxic waste products from the bloodstream through renal cell activity. The onset of renal dysfunction usually involves an interplay of factors such as inflammation, immune cell recruitment, and cell death. Compromised kidney function can result in the deposition of fibrous matrices that disrupt kidney tissue architecture and functionality (54, 55). In a normal steady-state environment, various innate and adaptive immune cells reside in the kidney, including dendritic cells, mast cells, macrophages, natural killer cells, NKT (natural killer T) cells, T cells, and B cells (Figure 3). These cells carry out essential functions to maintain renal homeostasis (56).

For example, macrophages clear pathogens and cellular debris, while dendritic cells uptake and present antigens to activate lymphocytes. T cells collaborate with other immune cells to generate cytokines that protect the kidney and preserve its intrarenal environment (56).

Systemic immunological and autoimmune illnesses, such as complement diseases, immune complex-related seropathies, systemic autoimmunity, and vasculitis, frequently target the kidney, causing significant renal damage (57). Thus, immune cells play a crucial role in the pathogenesis of renal disease.

It has been shown that m6A methylation is involved in immune regulation in the renal immune microenvironment, and many immune responses are closely related to m6A regulators. For example, the m^6^A-binding protein YTHDF1 prolongs neoantigen-specific immunity through m^6^A methylation modification of mRNA. Antigen cross-presentation of CD8^+^ T cells is also closely associated with YTHDF1 (58). m^6^A “author” protein METTL3 regulates homeostasis and differentiation of mouse T cells (59). Most HLA (human leukocyte antigen) genes are closely associated with m^6^A regulators, while HLA-DR3, HLA-DR4, HLA-DR11 and HLA-DR15 promote or ameliorate renal damage in LN (60). When the kidney is damaged, a large number of macrophages are produced, and the resulting pro-inflammatory factors (including TNF-a and IL1b) keep spreading, which in turn induce kidney inflammation. And m^6^A was found to be closely associated with macrophage phenotype and dysfunction (61).

However, the relationship between m^6^A modifications and immune signatures remains to be elucidated, which may be related to the limitations of the detection technology tools. Samples used for RNA sequencing contain a very limited number of immune cells, which may lead to a decrease in the accuracy of the abundance of infiltrating immune cells at the time of detection (62). Thus, the role of immune cell m^6^A modification in regulating renal homeostasis and renal disease development is unclear.

### m^6^A in AKI

AKI is a disease with severe drop of excretory renal function, which is characterized by a sharp rise in blood creatinine levels and a sharp fall in urine output (63). The overall prevalence of AKI is relatively high, affecting between 8 and 16% of hospitalized patients, and can lead to serious short- and long-term complications if left untreated (64). Specially, over 50% of ICU patients suffered from AKI, which was significantly associated with increased mortality rates (65). The main pathological features of AKI are acute tubular necrosis (ATN), interstitial inflammation, collapsing glomerulopathy, and an absence of immune deposition (66).

Although AKI has historically been classified into three categories, recent advancements in AKI research have led to a more nuanced understanding of the various types of AKI. These include, but are not limited to hepatorenal, cardiorenal, nephrotoxic, and sepsis-related AKI. These subtypes of AKI are distinguished not only by the underlying pathological mechanisms that contribute to AKI, but also by the unique pathophysiological interactions among different organ systems. We next describe the mechanism of action between m^6^A and ischemia–reperfusion Injury (IRI) -induced AKI, cisplatin-induced AKI (CI-AKI), sepsis-associated acute kidney injury (SA-AKI).

## IRI-induced AKI

One of the chief reasons of acute kidney injury is IRI, a syndrome in which the injury increases instead after tissue ischemia restores blood flow. IRI can have negative effects in a clinical environment, such as decreased kidney graft survival and elevated client mortality (67–69). m^6^A mRNA changes have been observed in the heart, brain and kidney IRI in both *in vivo* and *in vitro* experiments (70–73). Inhibiting m^6^A methylation shields organs from the harm caused by IRI, which is assumed to be a major mechanism of m^6^A RNA alterations in organ damage.

**Figure 4:**
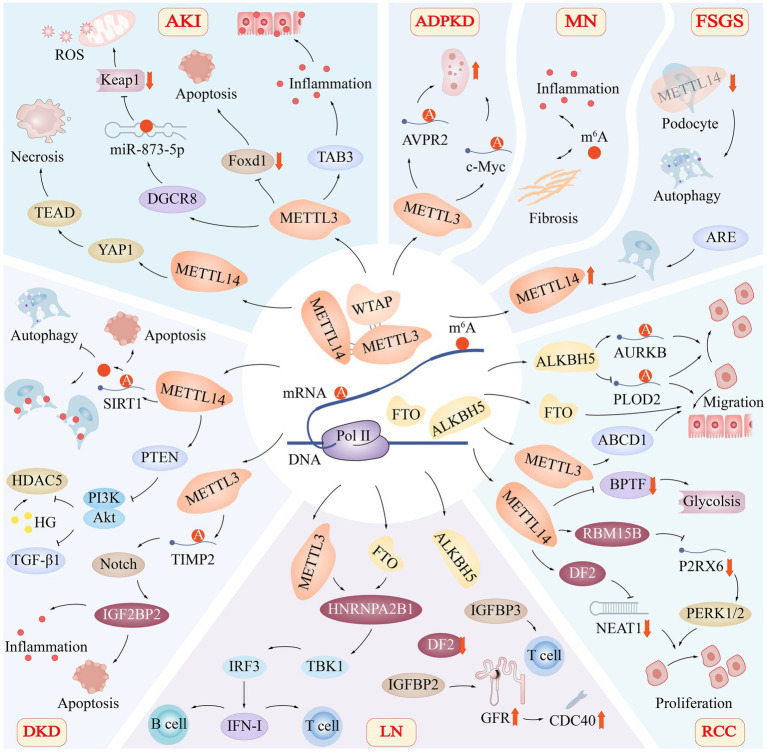
Role of m^6^A Modification in Renal Diseases. The m^6^A modification of RNA has been found to play a significant role in various renal diseases, including acute kidney injury (AKI), autosomal dominant polycystic kidney disease (ADPKD), membranous nephropathy (MN), focal segmental glomerulosclerosis (FSGS), diabetic kidney disease (DKD), lupus nephritis (LN), and renal cell carcinoma (RCC). The regulation of m6A modification involves critical subunit proteins such as METTL3, METTL14, WTAP, FTO, and ALKBH5, which have distinct roles in the progression of these diseases. Acute Kidney Injury (AKI): In AKI, METTL3 promotes TAB3 and DGCR8 expression while suppressing Foxd1 expression. Additionally, METTL14 targets the YAP1-TEAD axis, contributing to renal tissue necrosis. Autosomal Dominant Polycystic Kidney Disease (ADPKD): In ADPKD, METTL3 promotes cyst proliferation by increasing the methylation and translation of arginine-vasopressin receptor 2 (AVPR2) and c-Myc mRNA. Membranous Nephropathy (MN): In MN, m^6^A modification is involved in regulating key pathophysiological processes, including inflammation and fibrosis. Focal Segmental Glomerulosclerosis (FSGS): Knocking down METTL14 in podocytes in FSGS leads to improvements in glomerular function and mitigates podocyte injury by activating autophagy and suppressing apoptosis and inflammation. Renal Cell Carcinoma (RCC): In RCC, FTO, ALKBH5, and METTL14 co-regulate the migration of renal epithelial cells. METTL14 also targets metabolic pathways, such as glycolysis. Lupus Nephritis (LN): In LN, METTL3 and FTO-mediated methylation of RNA m^6^A regulate the activation of the TBK1-IRF3 pathway via heterogeneous nuclear ribonucleoprotein A2B1 (HNRNPA2B1), thereby promoting IFN-I production. Additionally, CDC40 is positively correlated with glomerular filtration rate (GFR), suggesting a potential protective effect. Diabetic Kidney Disease (DKD): In DKD, reducing the expression of the METTL14 gene prevents SIRT1 mRNA m^6^A from being degraded, promotes autophagy, reduces apoptosis and inflammatory responses, and protects injured podocytes. Moreover, METTL14 enhances phosphatase and tensin homolog (PTEN), leading to the inactivation of the PI3K/Akt pathway and decreased HDAC5 and TGF-β1 expression.

As crucial subunit proteins of the m^6^A methylation transferase complex, METTL3 and METTL14 have recently been revealed to have a role in the emergence of IRI. In several studies, the upregulation of METTL3 was observed in IRI patients and IRI-induced AKI mouse models, indicating that IRI can lead to raised METTL3 and RNA m6A modification levels (74). A reduction in the production of Agt (angiotensinogen), Ren (renin), Ace (angiotensin I-converting enzyme), and At1r (angiotensin II, type I receptor-associated protein), together with the separation of Ren + cells from the capillary wall, lead to altered renal perfusion (75). Forkhead box D1 (Foxd1) also contributes to maintaining proper kidney development and regulating the shape of the renal capsule (75, 76). Through controlling Foxd1, METTL3 may contribute to renal IRI. Fodx1’s m^6^A level is negatively regulated and its mRNA expression level is elevated when METTL3 is suppressed, which affects renal perfusion (74) (Figure 4).

Via its proliferative and pro-fibrotic actions during recovery, Yes-associated protein 1 (YAP1) is engaged in renal regeneration and fibrosis following acute IRI (77). Contrarily, Xu et al. discovered that METTL14 knockout HK-2 cells and METTL14 knockout animals had lower levels of YAP1 mRNA methylation in their kidneys, which led to reduced YAP1 protein translocation, indicating that METTL14 can control IRI via suppressing YAP1 (78) (Figure 4). The fact that METTL14’s *ex vivo* renal IRI protection was destroyed by peptide 17’s suppression of the YAP1- transcriptional enhanced associate domain (TEAD) pathway suggests that RNA methylation is necessary for the activation of the YAP1 and YAP1-TEAD pathways and is required for METTL14’s function in renal IRI. This shows that renal IRI development involves m^6^A RNA methylation control.

## CI-AKI

Cisplatin and other platinum derivatives have been widely adopted as chemotherapeutic agents for their ability to exert anticancer effects by interfering with cell DNA and mitochondrial function. Despite their effectiveness in tumor therapy, these drugs are burdensome to renal function and often induce nephrotoxicity, leading to an important complication known as AKI (79). Recent research indicates that apoptosis, necrosis, and inflammation jointly represent hallmarks of CI-AKI (80).

Necrotic apoptosis is the principal cause of proximal tubular cell death in cisplatin-induced nephrotoxic AKI. When any major determinant of the mixed-spectrum kinase structural domain-like protein (MLKL), receptor interaction protein 1(RIP1), or necrotic pathway receptor interaction protein 3 (RIP3) is suppressed, cisplatin-induced proximal tubular damage in mice is reduced.

One research indicated that m^6^A methylation primarily functions in numerous pathways linked to metabolism, cell death, and oxidation, namely, there is a strong association between m^6^A methylation and CI-AKI regulation (81). This was identified through examining the discrepancies in m^6^A methylation and RNA expression in renal tissues between normal mice and CI-AKI animal models. Another study discovered that cisplatin caused the death of mice renal tubular epithelial cells, overexpression of METTL3 and METTL14 of m^6^A in cells, and a notable reduction in the expression of the methyl scavenger enzyme FTO, leading to a much higher level of RNA m^6^A modification and aggravating renal damage (82). FTO, on the other hand, may lessen the symptoms of CI-AKI by lowering the amounts of p53 mRNA and translation to lessen the apoptosis it causes.

## SA-AKI

One of sepsis’s most prevalent and serious consequences is SA-AKI (83). In one study, mmu-miR-7,212-5p-Hmox1 in iron death has been demonstrated as a significant RNA regulatory pathway implicated in the pathophysiological process of SA-AKI in a mouse model of septic AKI. The promotion of AKI development by m^6^A RNA methylation alteration in SA-AKI has been shown (84).

A growing number of studies in recent years have revealed that ncRNAs, particularly microRNAs (miRNAs), are connected to AKI (85). Endogenous ncRNAs called miRNAs have a variety of biological roles, including the ability to inhibit protein translation and control the negative feedback regulation of target mRNAs via complementary binding to the 3′-UTR of the target gene (86).

It has been discovered that m^6^A alterations play a role in the processing of pri-miRNAs or the splicing of pre-miRNAs, which regulate miRNA production. METTL3 increases the quantity of pri-miRNAs m^6^A and the expression of miRNAs. The demethyltransferase ALKBH5 prevents pri-miR-193a from being processed. To operate more effectively, miRNA controls the binding and location of METTL3 and FTO (87, 88)(Figure 4). In AKI, many miRNAs are implicated in controlling programmed cell death. According to Jia et al., miRNA-21 overexpression prevented cell death by decreasing PDCD4 while miRNA-21 silencing enhanced cell death in septic AKI (89).

### m^6^A in CKD

CKD is a global health problem affecting millions of people worldwide, whose typical symptoms are proteinuria or a decreased glomerular filtration rate (90). Emerging evidence suggests that dysregulation of RNA modifications, specifically m^6^A, plays a crucial role in the pathogenesis of CKD.

Studies have demonstrated altered expression levels of m6A regulators, such as METTL3 and YTHDF2, in CKD models. METTL3 is responsible for adding m6A marks to mRNA, while YTHDF2 binds to m6A-modified transcripts and affects RNA stability and translation. Dysregulation of m6A regulators, including the upregulation of METTL3 and the downregulation of YTHDF2, has been linked to the activation of TGF-β signaling pathways and the subsequent development of renal fibrosis in CKD.

Renal fibrosis is a significant contributor to CKD, and obstructive nephropathy (ON) resulting from obstructive uropathy is a major cause of renal fibrosis (91).Transforming growth factor-beta 1 (TGF-β1) is a key mediator of renal fibrosis, and studies have shown that it stimulates the expression of the long non-coding RNA (lncRNA) metastasis-associated lung adenocarcinoma transcription product 1 (MALAT1) in ON patients. In TGF-β1-treated tubular epithelial cells, m^6^A modification exerts a regulatory role in positively regulating MALAT1 expression by engaging METTL3, suggesting the involvement of m6A modification in the MALAT1/miR-145/focal adhesion kinase (FAK) pathway of renal fibrosis (92). Simultaneously, METTL3 has been shown to promote the expression of miR-21-5p, which in turn activates the SPRY1/ERK/NF-κB pathway to induce inflammation and fibrosis (93).

Besides renal fibrosis, autophagy is also implicated in the progression of CKD (94). Individuals with CKD have decreased leukocyte m6A concentration and increased protein RNA demethylase FTO expression. The uremic toxin indoxyl sulfate modulates FTO and m6A modifications to induce cellular autophagy. By decreasing m6A levels through FTO’s RNA demethylation activity, indoxyl sulfate influences leukocyte autophagy. However, the effect of indoxyl sulfate on cellular autophagy can be prevented by suppressing m6A or knocking down FTO using 3-deazaadenosine (DAA), offering a new approach to treating CKD by targeting m6A RNA modification (95).

CKD include DKD, LN, autosomal dominant polycystic kidney disease (ADPKD), membranous nephropathy (MN), focal segmental glomerulosclerosis (FSGS), IgA nephropathy (IgAN), and we will next discuss the role that m^6^ A plays in these diseases.

## DKD

DKD is a common microvascular complication of diabetes mellitus that leads to kidney disease. Prolonged hyperglycemia in diabetic patients induces the buildup of extracellular matrix in the glomerular and tubulointerstitial compartments, thickening and hyalinization of the intrarenal vascular system, and gradual decline of renal function (96, 97).

Numerous investigations have demonstrated the close connection between m^6^A and the onset of diabetes, but it is unclear how m^6^A contributes to the etiology of DKD. Ling Jiang et al. identified METTL3-mediated m^6^A modification as a vital cause of podocyte damage in DKD (98). When METTL3 was overexpressed, m^6^A modification in the kidney of type 1 and type 2 diabetic mice was dramatically elevated, and inflammation and apoptosis in high glucose (HG)-stimulated podocytes were significantly enhanced, and yet these reactions were decreased dramatically when it was shut down. The mechanism is that METTL3 induced pro-inflammatory and pro-apoptotic effects by modulating Notch signaling via the m^6^A alteration of tissue inhibitor of metalloproteinase 2 (TIMP2) in a way reliant on IGF2BP2.

Sirtuin-1 (SIRT1) deacetylase, which is abundantly found in renal tissues, has a role in kidney disorders by controlling a number of cellular biological processes, including apoptosis, autophagy, and inflammation, which helps to lessen acute kidney damage and treat kidney fibrosis (99, 100). In a separate investigation, mice with DKD had higher levels of m^6^A RNA and higher levels of METTL14 expression in their kidneys. When the expression of the METTL14 gene was reduced, SIRT1 mRNA m^6^A was prevented from being modified and degraded, autophagy was encouraged, apoptosis and an inflammatory response were reduced, and the wounded foot cells were thus safeguarded (101) (Figure 4).

In the kidneys of diabetes people and animals, the levels of histone deacetylase 5 (HDAC5), a member of the class II HDAC subfamily, are greater than average. *In vitro* cultures of human renal tubular cell lines (HK2) with high glucose levels revealed enhanced HDAC5 expression. According to studies on the etiology of DN, the TGF-β1 pathway has a significant pro-fibrotic role (102, 103). In diabetic tubular cells, hyperglycemia enhanced HDAC5 expression, and through elevation of TGF-β1, HDAC5 overexpression led to epithelial-mesenchymal transition in renal tubular cells. The epithelial-mesenchymal transition of renal tubular cells was impacted by the overexpression of METTL14, a vital component of the m^6^A methyltransferase complex, which also elevated m^6^A RNA methylation levels, boosted phosphatase and tensin homolog (PTEN) leading to PI3K/Akt pathway inactivation, decreased HDAC5 and TGF-β1 expression (104)(Figure 4).

α-klotho is an anti-aging gene that has been found to prevent tubular and glomerular injury and attenuate DKD in diabetic mice. METTL14 can also exacerbate high-glucose-induced glomerular endothelial cell injury and DKD by mediating the m^6^A modification of α-klotho and increasing its methylation level, leading to downregulation of α-klotho expression (105).

## LN

Systemic lupus erythematosus (SLE) is a systemic autoimmune disease characterized by its heterogeneity and unknown etiology. Among its various manifestations, LN is the most frequent and severe, occurring in up to 60% of patients with SLE. LN can lead to significant kidney damage, with hematuria and proteinuria being common clinical features (106, 107).

m^6^A regulators in LN are connected to the immune microenvironment, according to some linked research, though the precise method by which m^6^A methylation is implicated in LN is unknown. Recent studies have revealed strong correlations between activated NK cells, immune responses, HLA genes, and m^6^A regulators in kidney tissues of patients with LN. Additionally, seven m^6^A markers [cell division cycle 5-like (CDC5L), cell division cycle 40(CDC40), heterogeneous nuclear ribonucleoprotein U (HNRNPU), nudix (nucleoside diphosphate linked moiety X)-type motif 21(NUDT21), poly(A) polymerase alpha(PAPOLA), polymerase (RNA) II (DNA directed) polypeptide B (POLR2B), and WW domain binding protein 4 (WBP4)] have been identified and implicated in the development and progression of LN. Among these markers, a positive correlation was observed between CDC40 and glomerular filtration rate (GFR), suggesting a potential protective effect (Figure 4). Conversely, CDC5L, HNRNPU, NUDT21, PAPOLA, POLR2B, and WBP4 were negatively correlated with GFR, raising the possibility that these genes may play a role in exacerbating renal damage in LN patients (108).

The expression of most m^6^A regulators in glomeruli differs significantly between healthy individuals and those with LN, indicating potential roles for these regulators in the pathogenesis of LN. Among these regulators, IGFBP3 plays an important role in maintaining a healthy immune system as a key m^6^A regulator, together with two key immune genes (CD14 and IDO1) (109).

It has been certified that urinary CD14 monocytes provide an effective biomarker for diagnosing LN (110). Meanwhile, IGFBP3, which controls somatic cell growth and proliferation, plays a critical role in healthy immune system maintenance, and supports the differentiation of naive CD8^+^ T cells. Additionally, free insulin-like growth factor-1 (IGF1) has been observed to have a convinced metabolic impact in patients with SLE. In MRL/LPR mice, the overexpression of IGF-1 and IGFBP2 in glomeruli has been associated with significant changes in renal morphology and function (111). Several studies have identified IGFBP2 as a promising biomarker for both SLE and LN. So IGFBP shows tremendous potential as a biomarker for autoimmune disorders (112).

Additionally, there is emerging evidence suggesting that ALKBH5 may serve as a key regulator in the pathogenesis of SLE (113). A potential link between reduced YTHDF2 expression and disease activity in SLE is also clarified (114).

Type I interferons (IFN-I) is a key player in the antiviral response of the innate immune system, and is also implicated in the pathogenesis of SLE. The methylation of RNA has been shown to play a crucial role in the production of IFN-I. For instance, m^6^A methylation of RNA mediated by METTL3 and FTO has been found to regulate the activation of TBK1-IRF3 pathway via heterogeneous nuclear ribonucleoprotein A2B1 (HNRNPA2B1), resulting in the promotion of IFN-I production (115) (Figure 4).

The above findings suggest that m6A methylation changes are important for the formation of LN. Hence, further analysis is necessary to elucidate the precise roles of m^6^A markers in the pathogenesis of LN and to develop more effective treatment strategies for this disease.

## ADPKD

ADPKD is a monogenic disorder characterized by the development of numerous expanding tubular-derived cysts, which affects 85% of affected individuals leading to kidney failure (116). In a seminal study, Ramalingam et al. reported that METTL3 and its target RNA modification, m^6^A, play a crucial role in the pathogenesis of tubular cyst growth in ADPKD. METTL3 promotes cyst proliferation by increasing the methylation and translation of arginine-vasopressin receptor 2 (AVPR2) and c-Myc mRNA, which in turn enhance cyclic adenosine monophosphate and c-Myc signaling (Figure 4). Moreover, m^6^A content is also increased in kidney tissues of patients with ADPKD, suggesting the clinical relevance of METTL3/m^6^A signaling in ADPKD patients (117).

## MN

MN is characterized by the inflammation and thickening of the glomerular basement membrane, which primarily arises from autoimmunity and the deposition of immune complexes in the kidney. The phospholipase A2 receptor (PLA2R) has emerged as a prominent target antigen in MN (118). Similarly to IgAN, miRNAs have been implicated in the onset, progression, and potential prevention of MN, although the precise mechanisms remain elusive. Differential expression analysis of miRNAs and mRNAs has revealed the presence of regulatory network genes that may contribute to the development of membranous nephropathy through various signaling pathways, including mTOR, PDGFR-β, LKB1, and VEGF/VEGFR (119).

Unfortunately, the specific connection between m^6^A modification and membranous nephritis has yet to be established. m^6^A modification is known to play a role in regulating key pathophysiological processes in the kidneys, such as inflammation, fibrosis, and immune responses (Figure 4). These processes could potentially be implicated in the pathogenesis of membranous nephritis. Consequently, further investigations into the relationship between m^6^A modifications and MN are warranted, with a focus on potential alterations in m^6^A regulators, target genes, and their impact on disease onset and progression.

## FSGS

FSGS is a non-specific lesion primarily affecting podocytes rather than a distinct disease entity. Its main clinical manifestation is variable proteinuria, with or without accompanying nephrotic syndrome (120). Given the crucial role of podocytes in the progression of proteinuric nephropathy, investigating the impact of m^6^A modification on podocyte injury has gained significant attention.

To investigate the role of m^6^A in podocyte injury, Lu et al. performed a dual luciferase reporter gene assay in cultured human foot cells stimulated with Adriamycin or advanced glycosylation end products (AGE). They observed that METTL14 was significantly increased in renal biopsy samples from patients with FSGS and diabetic kidney disease, as well as in cultured human foot cells treated with Adriamycin or AGE *in vitro*.

Interestingly, knocking down METTL14 in podocytes not only exhibited notable improvements in glomerular function but also mitigated podocyte injury by activating autophagy while concurrently suppressing apoptosis and inflammation (101) (Figure 4).

This groundbreaking discovery and further investigations in this area hold the potential to uncover novel therapeutic strategies targeting m^6^A regulatory pathways specifically in podocytes.

## IgAN

IgAN is the most prevalent primary glomerular disease globally and one of the leading causes of chronic renal diseases. Studies have shown that approximately 40% of patients progress to end-stage renal diseases within 20 years of diagnosis. A distinguishing characteristic in the histological diagnosis of IgAN is the presence of explicit or implicit IgA staining in kidney biopsies (121).

Emerging data suggests that at least four key processes contribute to the development of IgAN: hereditary increase in galactose-deficient circulating IgA1, circulating antibodies directed against galactose-deficient IgA1, formation of pathogenic IgA1-containing immune complexes, mesangial deposition of IgA1-containing immune complexes, cell activation, and initiation of glomerular injury (122).

Notably, studies have identified elevated expression of JCHAIN in interstitial cells, suggesting its potential involvement in the accumulation of IgA1 and the initiation of *in-situ* deposits within the renal tissue (123).

IgAN also has shown associations with miRNAs, implicating their potential role in disease pathogenesis. Notably, miR-133a, miR-133b, and miR-185 have been observed to facilitate IgA1 deposition, while miR-17-5p appears to be linked to thylakoid proliferation and endocytosis transport, both contributing factors to the appearance of IgAN (124). In the realm of human cancers, IGF2BP2, an m^6^A reader, demonstrates interactions with diverse RNA species, including miRNAs, mRNAs, and lncRNAs, thereby affecting cancer development and progression (125). Though the precise relationship between m^6^A modification and the underlying mechanisms of IgAN remains unclear, leveraging existing research as a foundation paves the way for future comprehensive investigations aimed at unraveling the mechanism of this disease and identifying novel therapeutic targets.

### m^6^A in RCC

RCC is a common form of malignancy in the urinary system, accounting for approximately 3% of adult cancer diagnoses worldwide (126). With over 270,000 new cases and 116,000 deaths each year, RCC is a significant global healthcare challenge (127). The tumor arises from the tubular epithelial cells of the renal parenchyma, and common clinical manifestations include hematuria, lumbar pain, and renal masses. Of all renal tumors, renal clear cell carcinoma (KIRC) represents the most prevalent histologic subtype, comprising 75% of all RCC cases (128). Furthermore, RNA alteration is crucial to the mechanism underlying the emergence of RCC.

The data supporting the association of m^6^A with the control of tumor features such carcinogenesis, proliferation, differentiation, invasion, and metastasis is presently mounting. For instance, ALKBH5 promoted cell proliferation in RCC by mediating m^6^A demethylation of mRNA AURKB (Aurora kinase B) to increase its stability (129) (Figure 4).

It has been demonstrated that abnormal m^6^A RNA alterations control cancer-related pathways and gene expression in ccRCC. Globally speaking, the m^6^A alterations in the current study appear to positively correlate with mRNA expression in ccRCC samples (130–132). Differentially methylated m^6^A sites in NADH dehydrogenase (ubiquinone) 1 alpha subcomplex, 4-like 2 (NDUFA4L2), Procollagen-Lysine, 2-Oxoglutarate 5-Dioxygenase 2 (PLOD2), NXPH family member 4 (NXPH4), Krüppel-like factor 11 (KLF11), Natriuretic peptide receptor 3 (NPR3), Uromodulin (UMOD), and ankyrin 3(ANK3) indicate that these genes are linked to ccRCC. The function of downstream m^6^A readers determines the precise role of m^6^A methylation on gene expression, so it may be beneficial to overexpress or knockdown key enzymes that have been modified with m^6^A in order to better understand m^6^A methylation-mediated cellular responses.

By bioinformatics analysis, METTL14 protein levels were significantly lower in renal tumor tissues than in paired normal tissues, and low levels of METTL14 enhanced the stability of bromodomain PHD finger transcription factor (BPTF), and accumulated BPTF constituted and reinforced enhancers or super-enhancers (SEs) activating enolase 2 (ENO2) and SRC proto-oncogene nonreceptor tyrosine kinase (SRC), leading to glycolytic reprogramming and triggering the aerobic glycolytic pathway, thus promoting RCC metastasis *in vitro* and *in vivo* (133). METTL14 can also inhibit the growth and metastasis of RCC by reducing long-stranded non-coding RNA nuclear enriched abundant transcript 1 (NEAT1_1) in a m^6^A- YTHDF2-dependent manner (134). Similar findings have been made in other research, which indicate that epithelial splicing regulatory protein 2 (ESRP2) ubiquitination is controlled by METTL14 to prevent the metastasis of clear cell RCC through Lnc-LSG1 m^6^A modification (135). By encouraging ESRP2 breakdown via the ubiquitination route, Lnc-LSG1 raises ESRP2 ubiquitination levels and prevents ccRCC metastasis. The anti-metastatic impact of Lnc-LSG1 on ccRCC cells was diminished as a consequence of METTL14, which decreased the binding of Lnc-LSG1 to ESRP2 protein via YTHDC1 and enhanced the stability of ESRP2 protein.

Patients with RCC often have a poor clinical prognosis and a high death rate due in large part to metastatic KIRC. In order to better understand the mechanism of cancer spread, lncRNAs are now thought of as a novel regulatory factor (136). They have been discovered to have a role in a number of crucial biological processes in malignancies. Two hub m^6^A-lncRNAs (LINC01820 and LINC02257) were found to be overexpressed in KIRC cell lines and were strongly related with a bad prognosis in KIRC patients in one research utilizing Cytoscape software to look for central m^6^A lncRNAs (137). These were, therefore, extremely potential therapy candidates for those with metastatic KIRC.

Certain RCC subtypes, including NONO-TFE3 translocated RCC (NONO-TFE3 tRCC), have also been discovered to have m^6^A involvement in their etiology in addition to renal clear cell carcinoma. As a tumor suppressor gene, TRAF3IP2 antisense RNA1 (TRAF3IP2-AS1) binds to poly ADP-ribose polymerase (PARP1) mRNA directly, boosting the m^6^A modification of PARP1 mRNA, attenuating PARP1 mRNA, and improving PTEN expression via binding miR-200a-3p, miR-153-3p, and miR-141–3p, therefore greatly decreasing PARP1 expression and suppressing NONO - TFE3 translocated RCC. On the reverse hand, NONO-TFE3-translocated RCC was encouraged when TRAF3IP2-AS1 was expressed at low levels (138).

## Potential therapeutic applications of targeting m6A regulators

An increasing number of kidney diseases, such as RCC, AKI and CKD, have been found to be associated with aberrant m^6^A. Today’s studies on the mechanisms of RNA methylation in renal diseases rely heavily on m^6^A, which has been found to affect normal physiological functions of the kidney by regulating the expression of target key genes (139, 140).

When m^6^A-related enzymes are abnormally expressed, they can interact with their downstream transcription factors to affect the mRNA synthesis process and promote or inhibit the development and progression of kidney disease. It was found that METTL3 overexpression of m^6^A in cells decreases Foxd1 content and causes apoptosis (74); METTL3 overexpression also leads to an increase in tgf-β-activated kinase 1 binding protein 3 (TAB3), resulting in inflammation and cell injury (10); METTL14 overexpression causes a decrease in YAP1, leading to reduced cell viability and thus acute kidney injury (71).

METTL3 is also present in chronic kidney disease. It positively regulates MALAT1 in TGF-β1-treated HK2 cells and affects the MALAT1/miR-145/FAK pathway in renal fibrosis, leading to renal fibrosis (102, 104). A significant decrease in the expression of the methyl scavenger enzyme FTO was accompanied by a significant increase in the level of RNA m^6^A modification and a concomitant increase in the amount of p53 mRNA, which in turn exacerbated renal damage (95).

METTL14 exacerbates high glucose-induced glomerular endothelial cell injury and DN by mediating m^6^A modification of α-klotho, increasing its methylation level and leading to downregulation of α-klotho expression (105). It can also affect the PTEN/PI3K/AKT pathway to increase HDAC5 and affect the epithelial-mesenchymal transition of renal tubular cells in DN (104). In contrast, METTL14 was significantly downregulated in RCC tissues (n = 580). Through the METTL14-YTHDF2-NEAT1_1 signaling axis, the growth and metastasis of RCC could be promoted (135).

The above-mentioned aberrantly expressed enzyme RNA methylation modification is a dynamic and reversible process. In renal diseases, reversing aberrant RNA methylation by targeting m^6^A regulators may delay the progression of renal disease. This suggests that targeting RNA m^6^A modification may be a novel strategy for the treatment of CKD and autophagy.

In recent years, researchers have made significant progress in developing targeted molecular inhibitors for m6A modifications. This approach is based on the observation that m^6^A regulators, such as METTL3, YTHDF1, and YTHDF2, are often dysregulated in tumor cells. These regulators are able to inhibit tumor cell proliferation, induce cancer cell death, and enhance immune response by increasing T cell transport and reducing immunosuppression (141).

Several inhibitors targeting FTO, such as rhodopsin, MO-I-500, and meclofenamic acid (MA), have been developed (142–144). Additionally, a highly potent and selective inhibitor of METTL3 and METTL14 called STM2457 has recently been identified. In preclinical models of acute myeloid leukemia, this inhibitor demonstrated significant anti-leukemic effects, suggesting that targeting m^6^A regulators holds promise for cancer therapy (145).

In addition to developing specific inhibitors of m6a regulatory factors, we may also want to start with epigenetic regulation, which plays a crucial role in gene expression and cellular function. The use of epigenetic inhibitors, such as DNA methyltransferase inhibitors or histone deacetylase inhibitors can modulate DNA methylation or histone modifications that may have an impact on kidney disease.

Given the complexity of kidney disease, we can first examine the enzymes that are aberrantly expressed by m6A modifications in patients, such as METTL3 and METTL14, which are expressed at elevated levels in patients with AKI (10, 78). Then, based on the results, we can make a diagnosis and administer a combination of treatments, such as using combinations of writing inhibitors, erasure modifiers, and reading protein modifiers, which may improve therapeutic efficacy.

While numerous potential therapeutic targets related to m6A methylation have been identified, there is a noticeable absence of clinical trials investigating the use of RNA methylation modifications in the treatment of renal diseases. Hence, there is a need for more targeted and meticulous clinical trials that utilize m6A methylation-associated enzymes for the treatment of renal diseases.

## Conclusion

In summary, chemical modifications of RNA have an important role in many processes of cellular life and in the development of renal systemic diseases. In many renal diseases, RNA modifications, especially m^6^A, play an important role. For example, elevated or decreased levels of METTL3, a core component of m^6^A methyltransferase, can have a significant impact on the pathological manifestations of AKI, renal IRI, and DN. In contrast, high expression of METTL14 can have damaging effects on renal podocytes. In RCC, chemical modifications of RNA play a role in its developmental mechanism. The degree of expression of various methylation complexes showed abnormalities in cancer samples. These modifications can regulate the fate of multiple diseases. Therefore, chemical modifications of RNA have the potential to be used for targeted therapies (Table 1).

**Table 1:** Possible drugs and small molecules in targeting m^6^A.

Possible drugs or small molecules	Target	Function	Reference
STM2457	METTL3	Inhibition of METTL3	(145)
UZH2	METTL3	Inhibition of METTL3	(146)
Sinefungin	METTL3	Inhibition of METTL3	(147)
Quercetin	METTL3	Inhibition of METTL3	(148)
AMF	METTL3	Inhibition of METTL3	(149)
RAD	METTL3	Inhibition of METTL3	(149)
SGI	METTL3	Inhibition of METTL3	(149)
JNJ	METTL3	Inhibition of METTL3	(149)
MEH	METTL3	Inhibition of METTL3	(149)
MHN	METTL3	Inhibition of METTL3	(149)
ECP	METTL3	Inhibition of METTL3	(149)
Elvitegravir	METTL3	Suppressed metastasis by directly targeting METTL3 and enhancing its STUB1-mediated proteasomal degradation	(150)
FB23	FTO	Inhibition of FTO activity	(151)
FB23-2	FTO	Inhibition of FTO activity	(151)
rhodopsin	FTO	Inhibition of FTO activity	(142)
MO-I-500	FTO	Inhibition of FTO activity	(143)
MA	FTO	Inhibition of FTO activity	(144)
R-2-hydroxyglutarate (R-2HG)	FTO	It binds competitively to FTO and inhibits its enzyme activity	(144)
Saikosaponin-d (SsD)	FTO	SsD directly targeted FTO, thereby increasing global m6A RNA methylation	(152)
FTO-43	FTO	Increase m^6^A and m^6^A_m_ levels	(153)
FTO inhibitor named 18,097	FTO	Bind the active site and selectively inhibit the demethylase activity of FTO	(154)
CHTB	FTO	Inhibit the demethylation activity of FTO and destroy the catalytic function of FTO	(155)
2-[(1-hydroxy-2-oxo-2-phenylethyl)sulfanyl]acetic acid (3)	ALKBH5	Inhibitor of ALKBH5	(156)
4-{[(furan-2-yl)methyl]amino}-1,2-diazinane-3,6-dione (6)	ALKBH5	Inhibitor of ALKBH5	(156)
Ena15	ALKBH5	Inhibitor of ALKBH5	(157)
Ena21	ALKBH5	Inhibitor of ALKBH5	(157)
MV1035	ALKBH5	Inhibitor of ALKBH5	(158)
Cucurbitacin B (CuB)	IGF2BP1	Direct targeting of IGF2BP1 blocks the recognition of m^6^A mRNA target by IGF2BP1 and induces apoptosis of cancer cells.	(159)
BTYNB	IGF2BP1	Specific inhibitor of IGF2BP1	(158)
JX5	IGF2BP1	Inhibitor of IGF2BP1	(160)
CWI1-2	IGF2BP2	Inhibition of IGF2BP2	(161)
Ebselen	YTHDF	Disrupt the interaction of the YTHDF m6A domain with the m6A-decorated mRNA targets	(162)

## Author contributions

ZH [2nd author], YW, and LaY were involved in the conception of the study. LuY, ZH [14th author], HC, LC, and YL were involved in writing the article. ZH [2nd author] and YW made and modified figures. BW, YF, MZ, JL, FP, and YM critically revised the manuscript. All authors contributed to the article and approved the submitted version.

## Glossary

3′ UTR: 3′ Untranslated region
5′ UTR: 5′ Untranslated region
Ace: Angiotensin I-converting enzyme
ADPKD: Autosomal dominant polycystic kidney disease
AGE: Advanced glycosylation end products
Agt: Angiotensinogen
AKI: Acute kidney injury
ANK3: Ankyrin 3
At1r: Angiotensin II, type I receptor-associated protein
ATN: Acute tubular necrosis
ASF/SF2: Alternative splicing factor/splicing factor 2
AVPR2: Arginine-vasopressin receptor 2
AURKB: Aurora kinase b
BPTF: Bromodomain PHD finger transcription factor
CCR4-Not: carbon catabolite-repression 4-not
CI-AKI: Cisplatin-induced AKI
CKD: Chronic kidney disease
CNV: Copy number variation
DAA: 3-deazaadenosine
DKD: Diabetic kidney disease
eIFs: Eukaryotic initiation factors
ENO2: Enolase 2
ESRP2: Epithelial splicing regulatory protein 2
FAK: Focal adhesion kinase
FSGS: Focal segmental glomerulosclerosis
Foxd1: Forkhead box d1
FTO: Fat mass and obesity-associated protein
GFR: Glomerular filtration rate
HDAC5: Histone deacetylase 5
HG: High glucose
HLA: Human leukocyte antigen
HK2: Human renal tubular cell lines
HNRNPA2B1: Heterogeneous nuclear ribonucleoprotein a2b1
IgAN: IgA nephropathy
IGF1: Insulin-like growth factor-1
IGF2BP1/2/3: Insulin-like growth factor 2 mRNA-binding protein 1/2/3
IFN-I: Type I interferons
IRI: Ischemia–reperfusion injury
KIRC: Renal clear cell carcinoma
KLF11: Krüppel-like factor 11
LN: Lupus nephritis
lncRNA: Long non-coding RNA
m^1^A: n1-methyladenosine
m^5^C: 5-methylcytosine
m^7^G: 7-methylguanosine
m^6^A: n6-methyladenosine
MALAT1: Metastasis-associated lung adenocarcinoma transcription product 1
METTL3: Methyltransferase-like protein
METTL14: Methyltransferase-like protein 14
METTL16: Methyltransferase-like protein 16
miRNAs: Micrornas
MGCS1: m^7^G-related cancer subtypes1
MLKL: Mixed-spectrum kinase structural domain-like protein
MN: Membranous nephropathy
NEAT1_1: Nuclear enriched abundant transcript 1
ncRNAs: Non-coding RNAs
NDUFA4L2: NADH dehydrogenase (ubiquinone) 1 alpha subcomplex, 4-like 2
NKT: Natural killer T
NPR3: Natriuretic peptide receptor 3
NXPH4: NXPH family member 4
ON: Obstructive nephropathy
PARP1: Poly ADP-ribose polymerase
PLA2R: Phospholipase A2 receptor
PLOD2: Procollagen-lysine, 2-oxoglutarate 5-dioxygenase 2
PTEN: Phosphatase and tensin homolog
RCC: RNA binding motif protein 15a/15b (RBM15A/RBM15B); renal cell carcinoma
Ren: Renin
RIP1: Receptor interaction protein 1
rRNA: Ribosomal RNA
SA-AKI: Sepsis-associated acute kidney injury
SAM68: SRC associated in mitosis of 68 Kd
SC35: Splicing component 35
SLE: Sirtuin-1(SIRT1); systemic lupus erythematosus
snRNA: Small nuclear RNA
SRC: SRC proto-oncogene nonreceptor tyrosine kinase
SRSF1/3: Serine/arginine-rich splicing factor 1/3
TAB3: TGF-β-activated kinase 1 binding protein 3
TEAD: Transcriptional enhanced associate domain
TGF-β1: Transforming growth factor-beta 1
TIMP2: Tissue inhibitor of metalloproteinase 2
TRAF3IP2-AS1: traf3ip2 antisense RNA 1
tRNA: Transfer RNA
UMOD: Uromodulin
VIRMA: vir like m^6^A methyltransferase associated
WTAP: Wilms tumor 1-associated protein
YAP1: Yes-associated protein 1
YTHDC1-2: YTH domain containing 1–2
YTHDF1-3: YTH m^6^A RNA binding protein 1–3
ZC3H13: Zinc finger CCCH-type containing 13
